# Deep learning, deeper relief: pipeline toward tailored analgesia for experimental animal models

**DOI:** 10.3389/fimmu.2025.1639881

**Published:** 2025-12-12

**Authors:** Luisa Barleben, Mareike Simon, Lisa Drees, Franziska Flohr, Christoph Jochum, Michela Di Virgilio, Frank Tacke, Sonja Bröer, Jana Wolf, Marina Kolesnichenko

**Affiliations:** 1 Charité– Universitätsmedizin Berlin, Corporate Member of Freie Universität Berlin and Humboldt- Universität zu Berlin, Department of Hepatology and Gastroenterology, Berlin, Germany; 2 Max Delbrück Center for Molecular Medicine, Berlin, Germany; 3 Mathematical Modelling of Cellular Processes, Max Delbrück Center for Molecular Medicine, Berlin, Germany; 4 Laboratory of Genome Diversification and Integrity, Max Delbrück Center for Molecular Medicine, Berlin, Germany; 5 Charité - Universitätsmedizin Berlin, Berlin, Germany; 6 Institute of Pharmacology and Toxicology, School of Veterinary Medicine, Freie Universität Berlin, Berlin, Germany; 7 Department of Mathematics and Computer Science, Freie Universität Berlin, Berlin, Germany

**Keywords:** NF-κB signaling, deep language, analgesia, amantadine, colitis

## Abstract

Effective pain management in animal models is crucial for maintaining ethical and scientific integrity. However, commonly used analgesics may affect immune responses and disturb signaling pathways, thereby potentially confounding the experimental outcomes. In mouse colitis models, opioids and non-steroidal anti-inflammatory drugs have been shown to interfere with the immune response and the activation of the central regulator of inflammation, the transcription factor nuclear factor kappa B (NF-κB). Here, we propose a tailored pipeline for the identification and the validation of analgesics with minimal off-target effects. This approach combines protein-centered relation extraction using deep language models and distant supervision via the Protein-Centered Association Extraction with Deep Language (PEDL^+^) together with an *in vivo* experimental validation with a NF-κB reporter mouse model that enables unambiguous visualization of direct NF-κB activity across different tissues. Our findings indicate that commonly used analgesics, such as tramadol and acetaminophen, not only interfere with immune cell recruitment and NF-κB activation but also skew the differentiation of epithelial stem cells into goblet cells, affecting epithelial functions even after short exposures. Conversely, the analgesics selected by our PEDL^+^-based workflow, such as piritramide, demonstrated no significant interference with NF-κB signaling. To validate our findings *in vivo*, we treated our NF-κB reporter mice with the analgesics selected by our computational pipeline. Amantadine demonstrated the least impact on the inflammatory responses and NF-κB activation. We then predicted and identified the signaling pathways that are impacted by amantadine treatment. In summary, our proposed pipeline facilitates a shift from one-size-fits-all analgesics to a precision medicine approach that considers the unique molecular interactions associated with each model.

## Graphical Abstract

**Figure:**
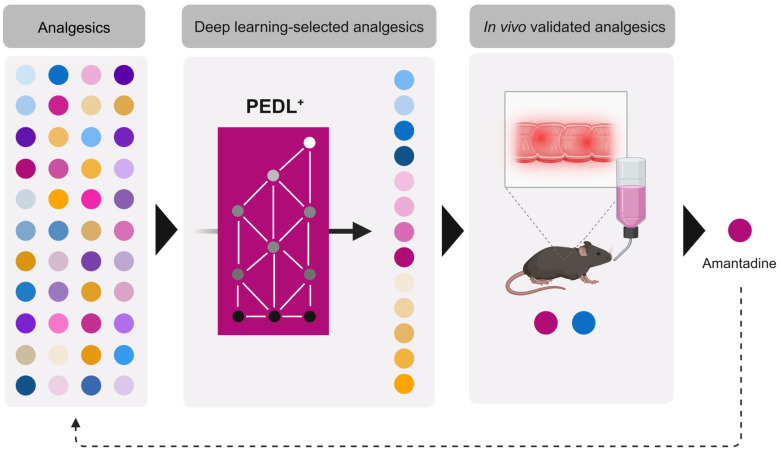

## Introduction

Inflammatory bowel diseases (IBDs) are characterized by chronic inflammation of the gastrointestinal tract and extraintestinal manifestations involving multiple organs (1). Due to the complexity of these multi-organ conditions, *in vitro* models remain insufficient, and animal models are indispensable to study the pathomechanisms of IBD. However, experimental animals often experience pain, which leads to suffering and physiological changes that can affect the experimental outcomes (2). Although there are numerous analgesics available for alleviating pain, these are often avoided due to their interference with the disease itself and the process studies—or, conversely, are administered despite their interference (3–5). For colitis models, buprenorphine (a µ-opioid receptor partial agonist), tramadol (a μ-opioid receptor agonist and a serotonin-norepinephrine reuptake inhibitor), paracetamol (an anti-pyretic), and metamizole (a non-acid non-opioid) are the most frequently used analgesics.

Analgesics can modulate or suppress the immune response and affect signaling pathways that regulate inflammation, such as nuclear factor kappa B (NF-κB) (3). The transcription factor NF-κB is deemed the master regulator of inflammatory responses: it controls the expression of over 400 genes, including those coding for the cytokines and chemokines involved in IBD (6). The activation state of NF-κB in leukocytes and intestinal epithelial cells (IECs) directly correlates with the severity of IBD (7). Numerous IBD therapies, ranging from corticosteroids to biologicals, either directly or indirectly suppress NF-κB (8, 9). It is therefore critical that analgesia should not interfere with NF-κB. However, numerous studies point to the contrary (10, 11). Thus, modeling IBD in mice that are given common analgesic drugs to alleviate the symptoms of colitis poses a great challenge in research reproducibility and accuracy. There is an urgent need to identify analgesics that do not interfere with the experimental outcomes.

An analgesic that has minimal interference with the disease in one experimental setting can have unforeseen perturbations in another model, thereby interfering with the interpretation of the results. Conversely, analgesics that are known to be inappropriate for certain experimental models, e.g., non-steroidal anti-inflammatory drugs (NSAIDs) for colitis studies, might be suitable for other models. There is therefore a clear need for tailored analgesia for different experimental models.

Due to the central role NF-κB plays in IBD and other immune-mediated pathologies, here, we aimed to identify analgesics that do not interfere with NF-κB signaling and also have minimal effects on immune functions. The search for the best-suited analgesic for a given model through indiscriminate testing of all the known compounds in animals is time-consuming and may be deemed unethical. In this paper, we propose an approach that facilitates the efficient identification of an optimal/custom-fit analgesic for experimental colitis in mice. We combined deep language model-based prediction with experimental validation to screen those compounds with the least detectable interference and subsequently confirmed our findings in our established NF-κB reporter mouse model (12, 13). The presence of NF-κB in the nucleus or even posttranslational modifications do not always indicate that the transcription factor is active. However, our reporter mouse model permits visualization of a transcriptionally active NF-κB. Importantly, this reporter system facilitates the detection of the hitherto neglected effect of analgesia on the functions of the intestinal epithelium (12, 13). We identified amantadine and piritramide as best suited for studies involving NF-κB. On the contrary, the commonly used tramadol and acetaminophen (paracetamol) have been predicted and confirmed to interfere with NF-κB and/or inflammation.

Our pipeline enables the identification of an analgesic that alleviates pain and shows the least interference with the disease in a murine chemical colitis model. Importantly, this approach can be extended to other experimental models to minimize animal suffering.

## Using protein-centered association extraction with deep language to identify analgesics with the least interference with NF-κB signaling

**Figure 1:**
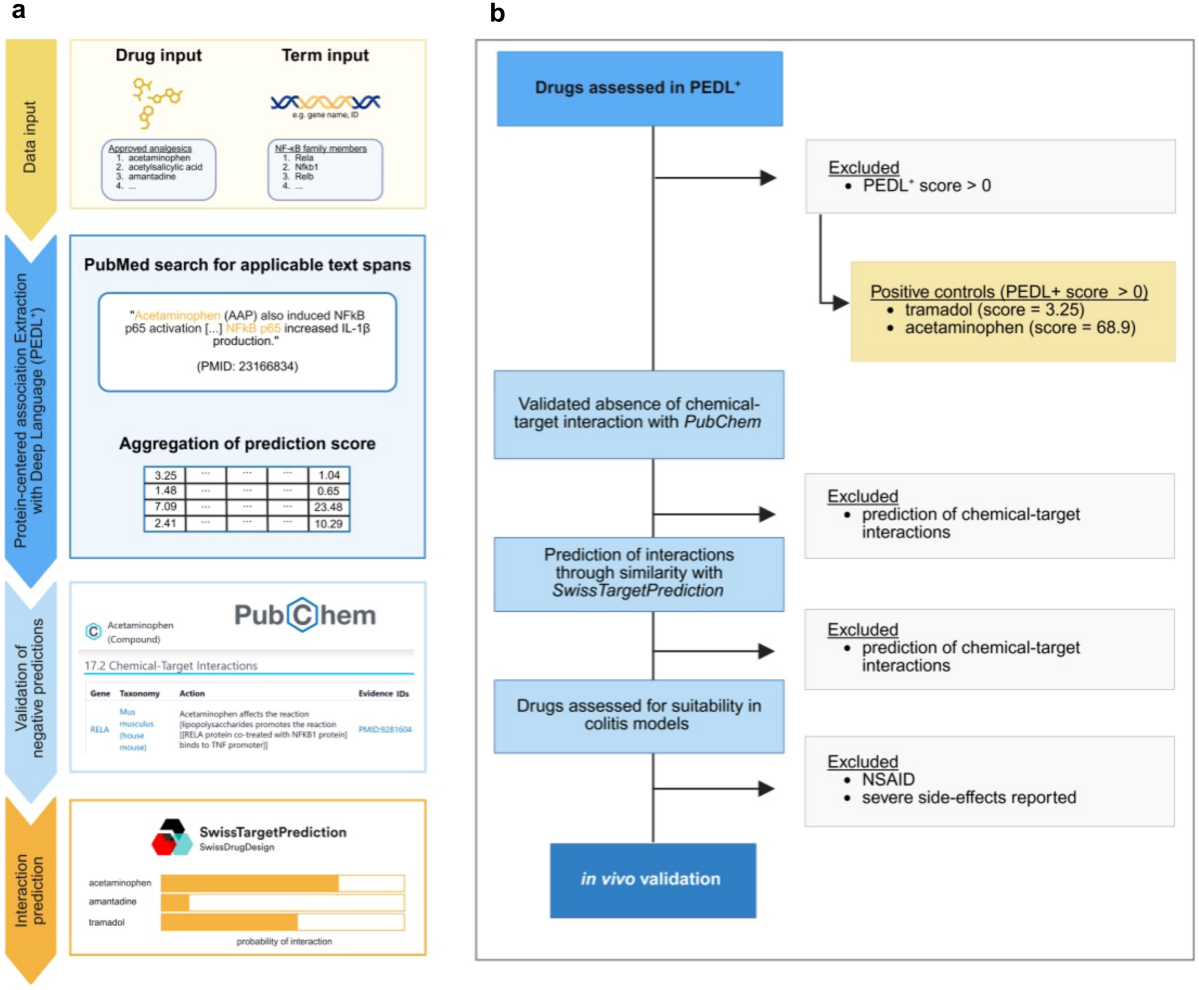
**(A)** Diagram of the workflow. Approved analgesics and NF-κB family members were used as input for Protein-Centered Association Extraction with Deep Language (PEDL+), which identified publications containing drug–gene interaction pairs and aggregated prediction scores for each analgesic. **(B)** Decision tree illustrating the inclusion and exclusion of all drugs assessed in PEDL+, which identified publications containing drug–gene interaction pairs. The prediction scores are aggregated for each analgesic. Analgesics without a hit in PEDL+ were cross-referenced against the PubChem database. The reasons for exclusion are listed on the *right*. *NSAID*, non-steroidal anti-inflammatory drug.

Protein-Centered Association Extraction with Deep Language (PEDL^+^) is a recently established approach that allows predicting protein–protein and protein–chemical associations from PubMed (14, 15). Evaluations in case studies have demonstrated that it can predict signaling events and relations that are missing in major pathway databases (14, 15). As NF-κB signaling is central to inflammation, we used this tool to predict and score the associations of NF-κB with known analgesics (**Figure 1A**) (15). These were obtained from national societies such as the Society of Laboratory Animal Science (GV-SOLAS) and from veterinary manuals such as the Merck Manual of Diagnosis and Therapy, or from university animal welfare databases (**Supplementary Table S1**). Known analgesics were chosen as, firstly, they have already shown efficacy in the alleviation of pain and, secondly, they can be readily implemented for animal research without extensive safety testing.

NF-κB comprises several family members, including RelA (p65), RelB, cRel, p50, and p52. In addition, upstream kinases such as IκB kinase (IKK) and other mediators dictate distinct transcriptomes and cell fates mediated by the signaling pathway (16, 17). These were included in PEDL^+^ for a comprehensive analysis of the pathway (**Supplementary Table S2**). We next analyzed the occurrence of both mouse and human terms (**Table 1**). A total of 17 analgesics received a score of 0, indicating no evidence of interference. Acetylsalicylic acid (an NSAID) had the highest score of 119.24 for interference with the NF-κB axis. The analgesics frequently used for colitis studies, i.e., tramadol and acetaminophen, received scores of 3.25 and 68.9, respectively. Both have been previously described as interfering with both NF-κB and the immune response. We therefore selected these commonly used drugs as positive controls.

**Table 1:** Results for all analgesics obtained with used in Protein-centered association Extraction with Deep Language (PEDL+) including Medical Subject Headings (MeSH) ID and Simplified Molecular Input Line Entry System (SMILES) notation. (-) No score calculated due to lack of publications.

analgesic	MeSH ID	SMILES	num_pub_ human	num_pub_ mouse	num_found_ human	num_found_ mouse	max_score_ human	max_score_ mouse	sum_score_ human	sum_score_ mouse
acetaminophen	D000082	CC(=O)NC1=CC=C(C=C1)O	22	62	27	84	0.95	0.96	21.45	68.9
acetylsalicylic acid	D001241	CC(=O)OC1=CC=CC=C1C(=O)O	223	69	793	143	0.97	0.96	694	119.24
amantadine	D000547	C1C2CC3CC1CC(C2)(C3)N	0	0	0	0	-	-	-	-
buprenorphine	D002047	CC(C)(C)C(C)(C1CC23CCC1(C4C25CCN(C3CC6=C5C(=C(C=C6)O)O4)CC7CC7)OC)O	0	1	0	1	-	0.9	-	0.9
butorphanol	D002077	C1CCC2(C3CC4=C(C2(C1)CCN3CC5CCC5)C=C(C=C4)O)O	3	0	6	0	0.89	-	4.78	-
carprofen	C007005	CC(C1=CC2=C(C=C1)C3=C(N2)C=CC(=C3)Cl)C(=O)O	0	1	0	3	-	0.88	-	2.45
cimixocib	C476594	COC1=C(C=C(C=C1)C2=C(N=CN2C3=CC=C(C=C3)S(=O)(=O)N)Cl)F	0	0	0	0	-	-	-	-
deracoxib	C471996	COC1=C(C=C(C=C1)C2=CC(=NN2C3=CC=C(C=C3)S(=O)(=O)N)C(F)F)F	0	0	0	0	-	-	-	-
dexmedetomidine	D020927	CC1=C(C(=CC=C1)C(C)C2=CN=CN2)C	66	48	138	107	0.96	0.96	111.73	88.09
firocoxib	C487384	CC1(C(=C(C(=O)O1)OCC2CC2)C3=CC=C(C=C3)S(=O)(=O)C)C	0	0	0	0	-	-	-	-
flunixin	C014557	CC1=C(C=CC=C1NC2=C(C=CC=N2)C(=O)O)C(F)(F)F	0	0	0	0	-	-	-	-
gabapentin	D000077206	C1CCC(CC1)(CC(=O)O)CN	3	1	4	2	0.92	0.66	2.85	1.23
grapiprant	C522837	CCC1=NC2=C(C)N=C(C)C=C2N1C1=CC=C(CCNC(=O)NS(=O)(=O)C2=CC=C(C)C=C2)C=C1	0	0	0	0	-	-	-	-
hydromorphone	D004091	CN1CCC23C4C1CC5=C2C(=C(C=C5)O)OC3C(=O)CC4	0	0	0	0	-	-	-	-
ibuprofen	D007052	CC(C)CC1=CC=C(C=C1)C(C)C(=O)O	28	9	74	10	0.97	0.94	65	8.49
ketamine	D007649	CNC1(CCCCC1=O)C2=CC=CC=C2Cl	43	26	63	38	0.97	0.97	52.71	31.73
ketoprofen	D007660	CC(C1=CC(=CC=C1)C(=O)C2=CC=CC=C2)C(=O)O	1	0	1	0	0.9	-	0.9	-
meloxicam	D000077239	CC1=CN=C(S1)NC(=O)C2=C(C3=CC=CC=C3S(=O)(=O)N2C)O	2	5	2	7	0.94	0.96	1.87	6.26
meperidine	D008614	CCOC(=O)C1(CCN(CC1)C)C2=CC=CC=C2	0	0	0	0	-	-	-	-
methadone	D008691	CCC(=O)C(CC(C)N(C)C)(C1=CC=CC=C1)C2=CC=CC=C2	7	3	15	6	0.94	0.91	11.68	4.86
morphine	D009020	CN1CCC23C4C1CC5=C2C(=C(C=C5)O)OC3C(C=C4)O	56	53	89	96	0.96	0.96	72.17	76.94
nalbuphine	D009266	C1CC(C1)CN2CCC34C5C(CCC3(C2CC6=C4C(=C(C=C6)O)O5)O)O	1	0	1	0	0.95	-	0.95	-
oxymorphone	D010111	CN1CCC23C4C(=O)CCC2(C1CC5=C3C(=C(C=C5)O)O4)O	0	0	0	0	-	-	-	-
phenylbutazone	D010653	CCCCC1C(=O)N(N(C1=O)C2=CC=CC=C2)C3=CC=CC=C3	0	0	0	0	-	-	-	-
piritramide	D010892	C1CCN(CC1)C2(CCN(CC2)CCC(C#N)(C3=CC=CC=C3)C4=CC=CC=C4)C(=O)N	0	0	0	0	-	-	-	-
pregabalin	D000069583	CC(C)CC(CC(=O)O)CN	3	2	4	2	0.94	0.92	2.97	1.78
remifentanil	D000077208	CCC(=O)N(C1=CC=CC=C1)C2(CCN(CC2)CCC(=O)OC)C(=O)OC	11	2	15	2	0.95	0.84	12.92	1.38
robenacoxib	C551524	CCC1=CC(=C(C=C1)NC2=C(C(=CC(=C2F)F)F)F)CC(=O)O	0	0	0	0	-	-	-	-
sufentanil	D017409	CCC(=O)N(C1=CC=CC=C1)C2(CCN(CC2)CCC3=CC=CS3)COC	7	5	24	6	0.97	0.95	20.44	4.34
tolfenamic acid	C009500	CC1=C(C=CC=C1Cl)NC2=CC=CC=C2C(=O)O	1	1	1	1	0.74	0.74	0.74	0.74
tramadol	D014147	CN(C)CC1CCCCC1(C2=CC(=CC=C2)OC)O	0	3	0	4	-	0.95	-	3.25
vedaprofen	C121784	CC(C1=CC=C(C2=CC=CC=C21)C3CCCCC3)C(=O)O	0	0	0	0	-	-	-	-
bisphosphonates	D004164	C(CC(O)(P(=O)(O)O)P(=O)(O)O)CN	9	2	11	2	0.95	0.91	9.32	1.72
diclofenac	D004008	C1=CC=C(C(=C1)CC(=O)O)NC2=C(C=CC=C2Cl)Cl	20	4	27	5	0.96	0.92	22.54	4.02
enflicoxib	C427706	C1C(N(N=C1C(F)(F)F)C2=CC=C(C=C2)S(=O)(=O)N)C3=C(C=C(C=C3)F)F	0	0	0	0	-	-	-	-
mavacoxib	C555097	C1=CC(=CC=C1C2=CC(=NN2C3=CC=C(C=C3)S(=O)(=O)N)C(F)(F)F)F	0	0	0	0	-	-	-	-
piroxicam	D010894	CN1C(=C(C2=CC=CC=C2S1(=O)=O)O)C(=O)NC3=CC=CC=N3	1	0	1	0	0.91	-	0.91	-
amitriptyline	D000639	CN(C)CCC=C1C2=CC= CC=C2CCC3=CC=CC=C31	6	6	31	9	0.96	0.92	23.79	7.58
dexketoprofen	C118296	CC(C1=CC(=CC=C1)C(=O)C2=CC=CC=C2)C(=O)O	0	0	0	0	-	-	-	-
etodolac	D017308	CCC1=C2C(=CC=C1)C3=C(N2)C(OCC3)(CC)CC(=O)O	0	0	0	0	-	-	-	-
tepoxalin	C073135	CN(C(=O)CCC1=NN(C(=C1)C2=CC=C(C=C2)Cl)C3=CC=C(C=C3)OC)O	1	0	1	0	0.95	-	0.95	-
pentazocine	D010423	CC1C2CC3=C(C1(CCN2CC=C(C)C)C)C=C(C=C3)O	1	1	1	1	0.89	0.68	0.89	0.68
indometacin	D007213	CC1=C(C2=C(N1C(=O)C3=CC=C(C=C3)Cl)C=CC(=C2)OC)CC(=O)O	36	30	46	40	0.98	0.95	37.81	31.79

Subsequently, we corroborated our findings using PubChem, an extensive database of chemicals that includes information on known interactions with biological targets (**Figure 1B**) (18). No evidence of chemical–target interactions was found for 16 of the 17 analgesics that received a score of 0 in PEDL^+^ (**Supplementary Table S3**). Finally, we used prediction of interaction through similarity with SwissTargetPrediction. By identifying proteins with established ligands that closely resemble the query structure, the targets of that structure may be predicted (19). SwissTargetPrediction integrates the similarity of shape and chemical structure into a combined target prediction score (20). We determined the targets related to NF-κB signaling for three of the 16 resulting analgesics using this tool (**Table 2**). In summary, we identified 13 analgesics that showed no evidence of interference with the NF-κB signaling pathway.

**Table 2:** List of analgesics without hits in PEDL+ and hits with SwissTargetPrediction ('1').

SwissTargetPrediction results		
analgesic	MeSH ID	Swiss Target Prediction
amantadine	D000547	0
cimicoxib	C476594	0
deracoxib	C471996	0
firocoxib	C487384	0
flunixin	C014557	0
grapiprant	C522837	1
hydromorphone	D004091	1
meperidine	D008614	0
oxymorphone	D010111	1
piritramide	D010892	0
robenacoxib	C551524	0
vedaprofen	C121784	0
enflicoxib	C427706	0
mavacoxib	C555097	0
dexketoprofen	C118296	0
etodolac	D017308	0

## PEDL^+^-selected analgesics show less interference than tramadol or acetaminophen with NF-κB signaling in the gut

Having no evidence of interference, however, does not prove lack of interference. We therefore sought to validate our findings *in vivo* and to determine whether the analgesics identified by PEDL^+^ as best suited also show the least interference with inflammation, immune cell composition in the gut, and NF-κB activity in epithelial cells. Of the 13 analgesics that showed no interference in PEDL^+^, PubChem, and SwissTargetPrediction, the majority fell into the NSAID class (which are known suppressors of inflammation) and were therefore dismissed. Of the three remaining analgesics, meperidine (pethidine) and piritramide belong to the opioid class, while amantadine belongs to the adamantane class. All three are already recognized as pain management medications (21). Due to the potential severe side effects of meperidine (22), we excluded it from further analyses.

To confirm the findings above, we administered *κEGFP* mice either amantadine or piritramide, or the positive controls (tramadol or acetaminophen), at the concentrations commonly used in veterinary practice during the duration of dextran sulfate sodium (DSS) treatment (22, 23). To reduce the number of animals experiencing colitis, we initially tested these analgesics under basal conditions, i.e., without DSS treatment.

**Figure 2:**
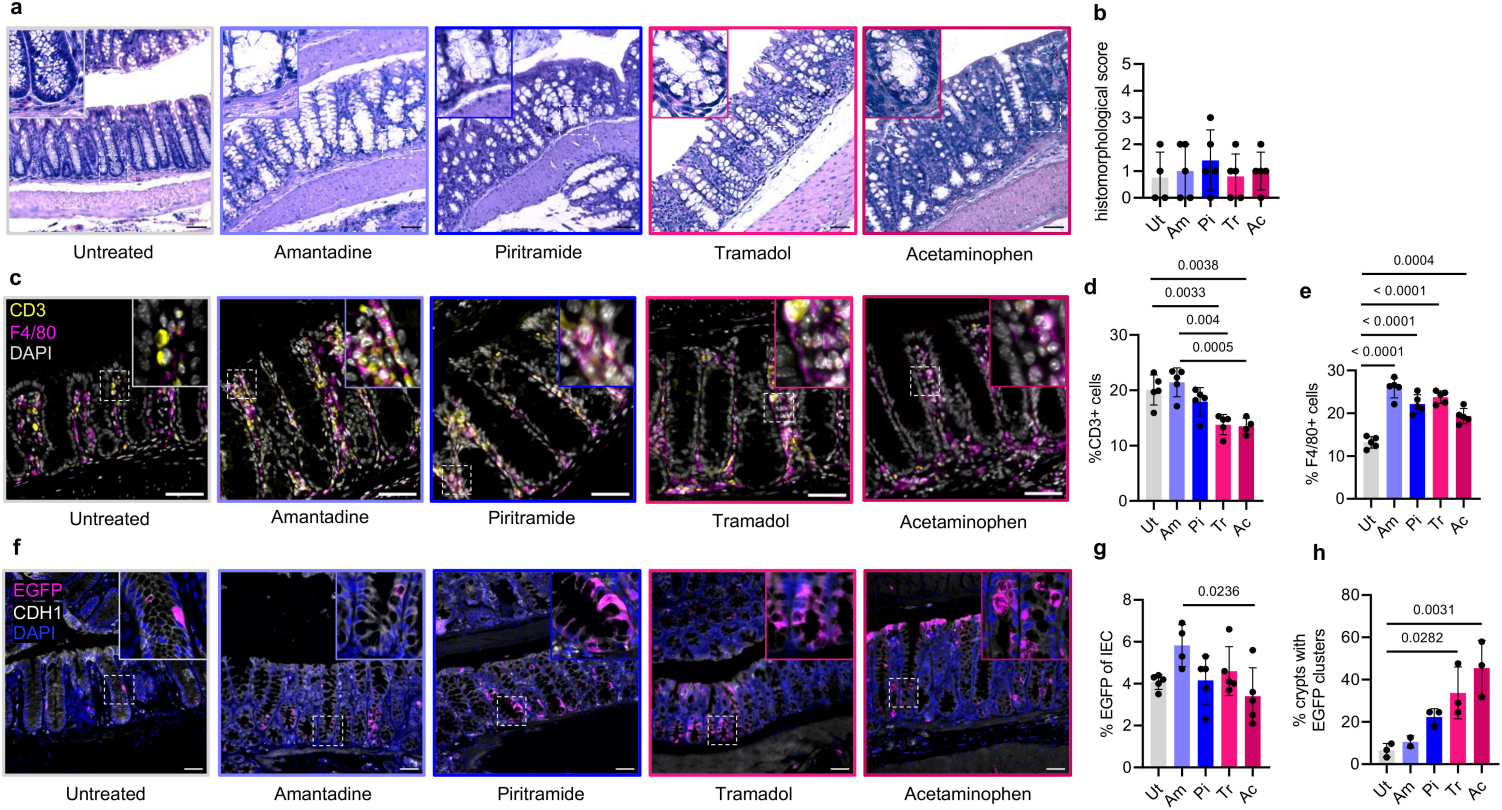
**(A)** H&E of the colon from mice treated with analgesics for 5 days or left untreated. Representative sections shown from at least *n* = 4 mice per group. *Insets* show magnified views of *dotted squares*. *Scale*, 50 μm. **(B)** Histomorphological score from **(A)**. *Ut*, untreated; *Am*, amantadine; *Pi*, piritramide; *Tr*, tramadol; *Ac*, acetaminophen. Analysis used one-way ANOVA with Tukey’s multiple comparison test. Only significant *p*-values shown horizontally [*F*(4, 19) = 0.3545, *p* = 0.838]. **(C)** Colon immunofluorescence (IF) staining for CD3 and F4/80 from mice treated as in **(A)** with at least *n* = 4. *Insets* show magnified views of *dotted squares*. *Scale*, 50 μm. **(D)** Quantitation of **(C)** shown as the percentage of CD3^+^ cells in the colonic lamina propria (*LP*). Analysis as in **(B)** [*F*(4, 19) = 11.37, *p* < 0.0001). **(E)** Quantitation of **(C)** shown as the percentage of F4/80^+^ cells in the colonic LP. Analysis as in **(B)** [*F*(4, 20) = 34.92, *p* < 0.0001]. **(F)** Colonic IF for EGFP and CDH1 from mice treated as in **(A)**. Representative sections shown from at least *n* = 4 mice per group. *Scale*, 50 μm. *Insets* show magnified views of *dotted squares*. **(G)** Quantitation of **(F)** shown as the percentage of EGFP+ cells in colonic CDH1+ intestinal epithelial cells (IECs). Analysis as in **(B)** [*F*(4, 19) = 3.06, *p* = 0.042]. **(H)** Quantitation of **(F)** shown as the percentage of colonic crypts with EGFP clusters (>4 EGFP+ cells in longitudinally cut crypt). Analysis as in **(B)** [*F*(4, 9) = 9.375, *p* = 0.0029].

To determine whether each analgesic on its own triggers inflammation, histomorphological scoring was performed (24). No significant increase in inflammation was detected with any of the analgesics tested (**Figures 2A, B**). To confirm these findings and further characterize the mucosal composition of leukocytes, the major types of mucosal leukocytes were visualized with immunofluorescence (**Figures 2C–E**). As previous studies have described changes in the number of macrophages and T lymphocytes (4, 25), we focused here on the F4/80^+^ and CD3^+^ cells in the mucosa. A significant change in the mucosal CD3^+^ T lymphocytes was detected only in mice treated with tramadol and acetaminophen (**Figure 2D**). The number of mucosal F4/80^+^ macrophages increased with all four analgesics tested (**Figure 2E**). These data are in accordance with previous studies showing that analgesics interfere with the immune response in the gut (4, 25).

The impact of the tested analgesics on epithelial NF-κB signaling was evaluated using enhanced green fluorescent protein (EGFP; as a marker of NF-κB activation) in E-cadherin (CDH1)+ IECs (**Figure 2F**). Although no significant change was observed in the average number of EGFP+ epithelial cells in the analgesic-treated groups compared with the untreated group (**Figure 2G**), a steep increase was detected in the percentage of colonic crypts containing EGFP clusters in both the tramadol and acetaminophen groups (**Figure 2H**). This suggests that NF-κB signaling was altered in the colon of the positive controls, but not significantly affected in the PEDL^+^-selected analgesics. Quantitative PCR (qPCR) on the bulk colon from amantadine- and piritramide-treated mice confirmed the minimal influence on the tested NF-κB target genes (**Supplementary Figure S2A**).

As chemically induced colitis in mice can impact inflammation in the distal small intestine (ileum) (26), we also examined the ileum to obtain baseline scores for the analgesic-treated mice (**Supplementary Figures S2B–H**). Histomorphological scoring showed no differences in inflammation or epithelial damage in the ileum of mice with any of the tested analgesics (**Supplementary Figures S2B, C**). The percentage of EGFP+ cells was significantly reduced only in the acetaminophen-treated group (**Supplementary Figures S2D, E**), whereas quantitation of the CD3^+^ T cells and F4/80^+^ macrophages revealed no significant differences in any group (**Supplementary Figures S2F–H**). Taken together, these findings indicate that the PEDL^+^-selected analgesics (amantadine and piritramide) interfered less with NF-κB signaling and the immune cell composition in the gut compared with the commonly used tramadol and acetaminophen.

## Analgesics selected by PEDL^+^ skew the differentiation of stem cells toward goblet cells

**Figure 3:**
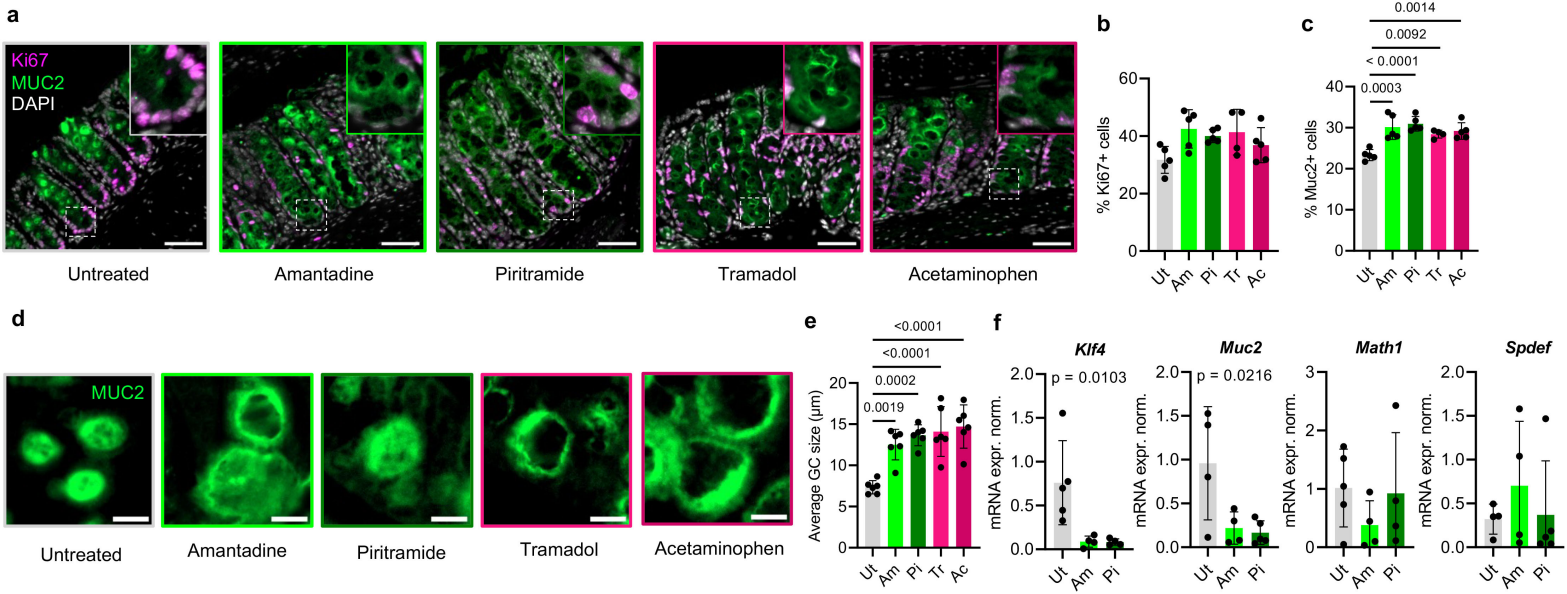
**(A)** Colon immunofluorescence (IF) staining for Ki67 and MUC2 from mice treated with analgesics for 5 days or left untreated. Representative sections shown from at least *n* = 4 mice per group. *Insets* show magnified views of *dotted squares*. *Scale*, 50 μm. **(B)** Quantitation of **(A)** shown as the percentage of Ki67^+^ cells in intestinal epithelial cells (IECs). *Ut*, untreated; *Am*, amantadine; *Pi*, piritramide; *Tr*, tramadol; *Ac*, acetaminophen. Analysis used one-way ANOVA with Tukey’s multiple comparison test. Only significant *p*-values shown horizontally [*F*(4, 19) = 2.790, *p* = 0.0560]. **(C)** Quantitation of **(A)** shown as the percentage of MUC2^+^ cells in IECs. Analysis as in **(B)** [*F*(4, 19) = 11.29, *p* < 0.0001. **(D)** Representative zoom-ins of **(A)** with an isolated MUC2 channel. *Scale*, 10 μm. **(E)** Quantitation of **(D)** shown as the average size of MUC2^+^ goblet cells (GCs). *Each dot* represents an average value of 10 measurements per image. *n* = 3 per group, two images per mouse assessed. Analysis as in **(B)** [*F*(4, 25) = 12.23, *p* < 0.0001. **(F)** Quantitative PCR (qPCR) analysis of the bulk colonic tissue of mice treated as in **(A)** with markers of epithelial cell type and differentiation. Normalized expression values (ΔΔCq) shown. Analysis as in **(B)**. Only significant *p*-values shown.

Previous studies have documented the interference of commonly used analgesics with the immune compartment (3). However, the effect of analgesia on the epithelium, and specifically on the cell fate decisions of stem cells, has not been explored. Hematoxylin–eosin (H&E) staining revealed prominent goblet cells in analgesic-treated mice (**Figure 2A**). We therefore stained mouse colons with mucin 2 (MUC2) and Ki67 to quantitate the goblet and proliferating stem cells, respectively (**Figures 3A–C**). We detected an increased total number of goblet cells, whereas Ki67^+^ cells were not affected by the analgesics (**Figures 3B, C**). Interestingly, the average size of the goblet cells increased significantly compared with the untreated controls (**Figures 3D, E**). To determine whether the analgesics interfered with the epithelial cell function or with differentiation, we performed qPCR on the bulk colon and analyzed the expression of the distinct markers of IEC types and their precursors. The expression of alkaline phosphatase (*Alpi)*+, chromogranin A (*ChgA)*+, and doublecortin-like kinase 1 (*Dclk1)*+, which mark enterocytes, enteroendocrine, and tuft cells, respectively, did not significantly change in the gut of mice treated with amantadine or piritramide (**Supplementary Figure S3A**). However, both *Muc2* and the goblet cell precursor *Krüppel-like factor 4* (*Klf4)* were significantly decreased in the PEDL^+^-selected analgesic groups when compared with the controls (**Figure 3F**). In the ileum, both piritramide and tramadol resulted in a reduced percentage of Ki67^+^ proliferating cells (**Supplementary Figures S3B, C**). Treatment with piritramide, but not with the other analgesics, decreased the total number of MUC2^+^ cells (**Supplementary Figure S3D**). In summary, these data demonstrate that, although the PEDL^+^-selected analgesics had a minimal effect on NF-κB signaling, they can interfere with the differentiation of the secretory epithelial cells in the colon. As piritramide also altered the cell composition in the small intestine, only amantadine was selected for further experiments.

## Amantadine as the best-suited analgesic for DSS colitis studies

**Figure 4:**
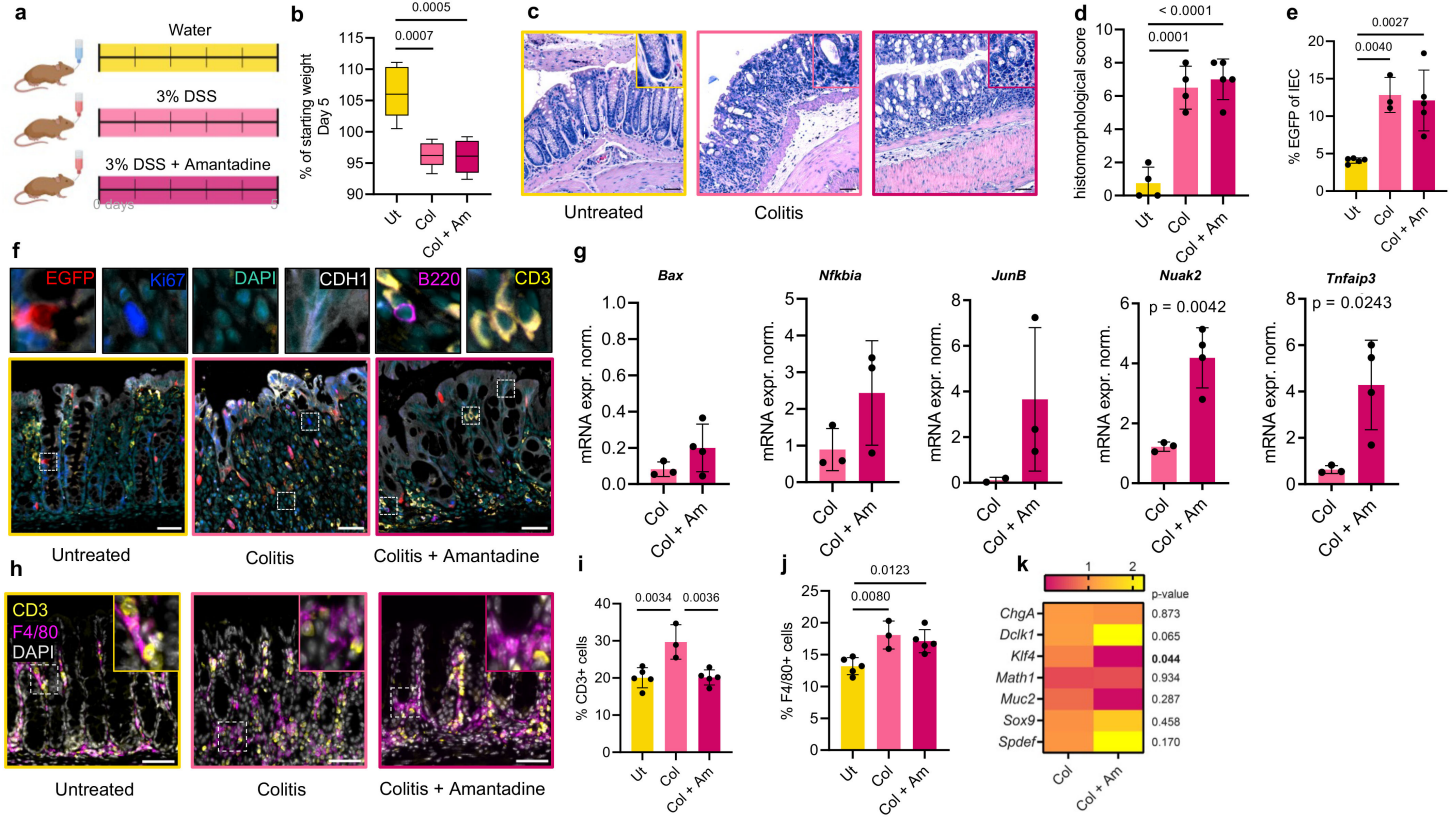
**(A)** For colitis induction, mice received 3% dextran sulfate sodium (DSS), with or without treatment of amantadine for 5 days. Control mice were left untreated. **(B)** Weight change of mice treated as in **(A)**. *Ut*, untreated; colitis, *Col*; *Col + Am*, colitis + amantadine. Average weight on day 5 shown as a percentage of the starting weight. *n* = 5 mice per group. Analysis used one-way ANOVA with Tukey’s multiple comparison test. Only significant *p*-values shown horizontally [*F*(2, 12) = 17.89, *p* = 0.0003]. **(C)** H&E of the colon from mice treated as in **(A).** Representative sections shown from at least *n* = 4 mice per group. *Insets* show magnified views of *dotted squares*. *Scale*, 50 μm. **(D)** Histomorphological score from **(C)**. Analysis as in **(B)** [*F*(2, 10) = 36.79, *p* < 0.0001]. **(E)** Quantitation of immunofluorescence **(IF)** from mice treated as in **(A)**. EGFP+ percentage from CDH1^+^ epithelial cells shown. *n* = 3 mice per group. Analysis as in **(B)** [*F*(2, 10) = 13.78, *p* = 0. 0013]. **(F)** Colon multiplex IF staining for EGFP, Ki67, CDH1, B220, and CD3 from mice treated as in **(A).***n* = 2. *Scale*, 50 μm. **(G)** Quantitative PCR (qPCR) analysis of the bulk colonic tissue of mice treated as in **(A)** with targets of NF-κB. Normalized expression values (ΔΔCq) shown. Unpaired *t*-test. **(H)** Colon IF for CD3 and F4/80 from mice treated as in **(A)**. *n* = 3. *Scale*, 50 μm. **(I)** Quantitation of **(H)** shown as the percentage of CD3^+^ cells in the colonic lamina propria (*LP*). Analysis as in **(B)** [*F*(2, 10) = 11.87, *p* = 0.0023]. **(J)** Quantitation of **(H)** shown as the percentage of F4/80^+^ cells in the colonic LP. Analysis as in **(B)** [*F*(2, 10) = 9.752, *p* = 0.0045]. **(K)** qPCR analysis of the bulk colonic tissue of mice treated as in **(A)** with differentiation markers. Normalized expression values (ΔΔCq) shown. Analysis as in **(G).** Significant *p*-values <0.05 shown in *bold*.

We next sought to validate amantadine as the best-suited analgesic for colitis studies in mice. We administered amantadine to mice at the same time as DSS. We treated mice for 5 days with 3% DSS in drinking water, which is known to produce moderate colitis and weight loss (**Figure 4A**). The Mouse Grimace Scale (MGS) (27) was used to validate amantadine as a functional analgesic (**Supplementary Figure S4A, B**). After 5 days, both the DSS-treated mice and those treated with DSS and amantadine lost equivalent amounts of weight compared with the untreated mice (**Figure 4B**). Similarly, histomorphological scoring of colitis revealed no significant change between the two groups (**Figures 4C, D**). Therefore, amantadine did not interfere with the development or the severity of DSS-induced colitis.

To determine how amantadine affected the activation of NF-κB and the composition of the mucosal leukocytes in colitis, we performed multiplex staining against EGFP and the markers of the major immune cell types in the gut (**Figures 4E, F**). As expected, the percentage of epithelial EGFP+ (with NF-κB activation) cells increased nearly threefold upon DSS treatment compared with the untreated group. Importantly, both the DSS and the DSS plus amantadine groups showed similar increases, indicating that amantadine does not suppress the activation of NF-κB in colitis (**Figure 4E**). Analysis of the NF-κB target genes in the bulk colon using qPCR revealed minimal impact on signaling (**Figure 4G**), with only two genes affected.

Quantitation of the mucosal immune cells showed an increase in both CD3^+^ T leukocytes and F4/80^+^ macrophages following colitis induction (**Figures 4H–J**). The decreased number of CD3^+^ lymphocytes after amantadine treatment suggests suppressed recruitment of lymphocytes (**Figure 4I**). As a manifestation of colitis induction, Ki67^+^ proliferating cells and MUC2^+^ goblet cells decreased after 5 days of treatment (**Supplementary Figures S4C–E**), as expected. Mice that additionally received amantadine showed an increased number of mature MUC2^+^ goblet cells (**Supplementary Figure S4E**), which, given that the severity of colitis was not affected, could be due to changes in differentiation (**Figure 3C**). In accordance with this observation, amantadine treatment resulted in reduced mRNA levels of the secretory cell precursor *Klf4* (**Figure 4K**).

We conducted additional analysis of the ileum tissue from DSS- and DSS plus amantadine-treated mice (**Supplementary Figure S4F–L**). No significant changes were observed between the two groups: following colitis induction, both groups showed similar histomorphological scores, percentages of EGFP+ epithelial cells, and number of F4/80^+^ macrophages and CD3^+^ lymphocytes in the lamina propria (**Supplementary Figures S4F–L**). No interference was observed in the small intestine of mice receiving amantadine together with DSS.

Although our approach facilitated the selection of analgesics that do not interfere with colitis or NF-κB signaling, it did not rule out interference with other signaling processes. Therefore, we then reversed part of the approach to identify signaling pathways potentially affected by amantadine. A total of 26 genes (**Supplementary Table S5**) affected by the analgesic were identified in PubChem. Gene set enrichment analysis revealed IL6/JAK/STAT signaling and interferon gamma response as the dominant pathways affected (**Supplementary Figure S4M**). To experimentally validate the prediction, we performed qPCR of the bulk RNA from the gut of amantadine-treated mice. The expression of interferon alpha/beta receptor 1 (*Ifnar1*) and oligoadenylate synthase 2 (*Oas2*) was activated by amantadine, confirming interference with both pathways.

In summary, of the analgesics assessed, amantadine had the least effect on the development or severity of colitis. NF-κB activation was comparable in both the colon and ileum in the group treated with the analgesic and in the DSS-only group. Nonetheless, amantadine promoted the accumulation of goblet cells, thereby altering the composition of the intestinal epithelium.

## Discussion

Analgesia alleviates unnecessary suffering in experimental animals and prevents changes associated with pain that could impact experimental readouts. Nonetheless, common analgesics often interfere with the cellular processes studied. Golusda and colleagues argued against the use of analgesia in its current forms (3). Here, we presented a novel approach to identifying the best-fitting analgesics for animal studies by combining deep language-based prediction with experimental validation. Our pipeline facilitated the detection of analgesics with the least interference with the signaling pathways studied. We showed that the analgesics commonly used to alleviate pain in experimental colitis—tramadol and acetaminophen—interfered with the inflammatory response and with NF-κB and should therefore be avoided in models of chemically induced colitis. Amantadine and piritramide, in contrast, were predicted and experimentally validated as the best-fitting analgesics for DSS colitis. PEDL^+^ has the potential to guide analgesic selection in experimental models beyond chemically induced colitis, enabling pathway-specific drug selection.

Colitis research has traditionally focused on the roles of immune cells, and accordingly, studies examining the side effects of analgesia have primarily investigated its impact on leukocyte function and recruitment (3). Although there are isolated reports that described disturbances in epithelial macroautophagy following NSAID treatment (28), our study, to our knowledge, is the first to demonstrate that commonly used analgesics can interfere with the differentiation of secretory epithelial precursors, leading to an accumulation of goblet cells. Reduced RNA expression of the secretory precursor genes such as *Klf4* can indicate the increased rate of differentiation and accumulation of only mature goblet cells. Indeed, the goblet cells in mice treated with analgesics were significantly larger in size with larger mucin stores, indicative of mature cells (29).

We have previously shown that epithelial-specific suppression of NF-κB promotes goblet cell expansion at the expense of other secretory lineages, supporting the idea that the altered differentiation in analgesic-treated mice may result from the direct inhibition of NF-κB signaling within IECs (13).

Some opioids are known to interact with Toll-like receptor 4 (TLR4) (30), which is expressed on subsets of IECs and may contribute to inflammation-related epithelial remodeling. In parallel, amantadine, an NMDA receptor antagonist (31), has been shown to target the NMDA receptors present on intestinal epithelial progenitors, where it may influence stem cell behavior and differentiation. Another intriguing possibility is the involvement of the gut–brain axis through either neural pathways—such as vagal efferent signaling—or hormonal mechanisms involving enteroendocrine mediators that then lead to changes in the differentiation of intestinal stem cells. In the future, the elucidation of these pathways will be essential for refining analgesic strategies that preserve epithelial homeostasis while minimizing interference with the intestinal biology in experimental models.

A notable strength and a limitation of our study is the use of compounds already tested as potential pain medications for the PEDL^+^ screen. Although deep learning can predict compounds not currently used as analgesics and therefore expand the repertoire of the currently used pain medications, it was not used here: *de novo* identification of analgesics would require extensive testing and validation and is beyond the scope of the current study. Instead, we focused on screening the existing analgesics and off-label pain medications that can be readily implemented in preclinical colitis models. Of note is that although amantadine is not considered a primary analgesic in Europe (EU regulation 2022/1255), it can be used as an adjunct treatment for pain. Amantadine is thought to act through increasing the extracellular dopamine levels and NDMA antagonism (32). Indeed, it is best known for the treatment of Parkinson’s disease and, previously, influenza. Nonetheless, it is important to explore its efficacy as a stand-alone analgesic and to better characterize its mode of action in future studies.

The selection of known compounds for the PEDL^+^ screen may not identify the “perfect” analgesic; however, it allows for the selection of the best-fitting compound from a pool of drugs with known safety profiles and proven analgesic efficacy. Moreover, the PEDL^+^-driven workflow facilitates drug repurposing, offering significant advantages in terms of reduced development time and costs compared with conventional drug discovery pipelines.

In conclusion, our findings emphasize the necessity of refining analgesic use in animal research to balance ethical considerations with scientific robustness. Identification of the best-fitting analgesics, such as amantadine for DSS-induced colitis, validates the effectiveness of this pipeline. Nonetheless, care should be taken to administer analgesics only when their benefit outweighs the drawbacks associated with their administration. For example, pain is often an integral part of diseases, including of IBD, that are modeled in mice. Therefore, it is important to determine when a reduction of pain permits more accurate mechanistic insights and when, on the contrary, analgesia creates additional confounding variables.

Whether all mice experience pain after DSS treatment is not clear: for example, some studies have failed to detect pain using the MGS in DSS-treated mice (33). The differences between studies could be due to the mouse strains used, the exact experimental conditions (including the DSS type), the facilities, and the specific methodology for detection. In our study, only a number of DSS-treated mice showed signs of pain according to the MGS. Therefore, we suggest that analgesia should be administered only when necessary, i.e., to mice showing signs of pain. Although this can introduce some confounding variables, this plan of action would be more in line with the situation in the clinic, where patients with IBD receive pain medication after and not before they feel pain.

By reducing the experimental variability and confounding effects introduced by analgesics, our approach contributes to the improved reproducibility and reliability in preclinical IBD research. Moreover, the broader applicability of this pipeline to other disease models highlights the potential of PEDL^+^ as a generalizable tool for optimizing analgesic selection in experimental animal studies. However, caution is warranted in the use of even optimized analgesics as unforeseen side effects may arise due to their extensive influence, as evidenced by the effects of analgesics on epithelial differentiation.

## Methods

### PEDL^+^-based pipeline

A local version of PEDL^+^ was applied to all relevant NF-κB genes for mouse and human, given by their NIH Gene IDs, and a list of the selected analgesics, given by their Medical Subject Heading (MeSH) IDs (**Supplementary Tables S1**, **S2**) (15). PEDL^+^ was used to identify publications from PubMed with drug–gene interaction pairs for these lists and to report the number of publications, the number of statements found, and the maximal score and the sum of all scores per drug for mouse and human genes, respectively. For those analgesics where PEDL^+^ had no hits, we additionally checked whether interactions are reported in PubChem (**Supplementary Table S3**) (18). The “Chemical–Target Interactions” list was downloaded and compared with the list of NF-κB genes. Subsequently, SwissTargetPrediction (34) was used to predict the targets for those analgesics that showed no interaction with NF-κB in PEDL^+^ and PubChem. Simplified Molecular Input Line Entry System (SMILES) structures were used as the input and *Mus musculus* selected as species. The results were downloaded and compared with the list of NF-κB genes. All targets with a probability >0.1 were considered as positive results. The analysis was performed in R.

### Mice

*B6-Tg(k-EGFP) 3Pt/Rsu* (text: *κEGFP*) mice were bred and received treatment at the Max Delbrück Center for Molecular Medicine in Berlin-Buch. The mice were housed under specific pathogen-free conditions with a 12-h light/12-h dark cycle, a room temperature of 22 ± 2°C, and humidity of 55% ± 10%. In addition, the mice received standard rodent chow, water *ad libitum*, and enrichment items. Genotyping and phenotyping were performed to confirm EGFP insertion (**Supplementary Table S4A**). For all experiments, randomized, age-matched male littermates between 8 and 24 weeks of age received treatment. The use of male mice allowed better comparison of our results with already published data (Chassaing, 2014 #186). Five mice were treated with either 2 mg/kg amantadine or 0.5 mg/ml tramadol or 3.5 mg/ml acetaminophen in the drinking water, or were left untreated or received an intraperitoneal (i.p.) injection of 10 mg/kg piritramide (35). Injection was performed by an experienced veterinarian on days 0 and 3. To induce colitis, male mice were given 3% DSS (colitis grade MFCD00081551; MP Biomedicals, Irvine, CA, USA) in their drinking water for 5 days. A second group of male mice received the same DSS treatment, along with 2 mg/kg amantadine throughout the entire duration. To ensure consistent drinking habits and to monitor the symptoms in both groups, the weight and water consumption of mice were monitored throughout the DSS treatment. All animal work, including experiments, was approved by the Berlin Landesamt für Gesundheit und Soziales (LaGeSo) (ethics approval nos. G0092/18 and G0175/23).

Tetrachloride injection produces an acute, sharp pain, which makes it easier to detect than any other symptoms caused by DSS. We therefore used this as a positive control for the MGS test.

### Mouse tissue processing

Mice were sacrificed at indicated times via cervical dislocation and immediately processed. The distal colon and the ileum were washed with phosphate-buffered saline (PBS), cut longitudinally, and fixed overnight in formalin as a “Swiss roll.” The tissues were then dehydrated by immersion in 70% ethanol (EtOH) overnight, followed by 30 min in 80% EtOH, 30 min in 90% EtOH, 60 min and another 30 min in 96% EtOH, 60 and 30 min in 100% EtOH, and 15 min in a 1:1 mixture of 100% EtOH and Roti Histol (cat. 6640; Carl Roth, Karlsruhe, Germany). Finally, the tissues were immersed in Roti Histol for 60 and 30 min. The intestinal tissues were embedded in paraffin (cat. X881; Paraplast Plus, Leica, Wetzlar, Germany) at 60°C overnight, solidified, and stored at 4°C. Sections of 4 µm were cut with a microtome (HM 355S; Thermo Scientific, Waltham, MA, USA), transferred into a slide, and dried at 37°C overnight. Proximal sections of the colon and the ileum were snap-frozen in liquid nitrogen for bulk RNA isolation.

### Quantitative PCR

RNA was isolated using TRIzol Reagent (cat. 15596018; Invitrogen, Carlsbad, CA, USA), and cDNA was synthesized with the iScript kit (cat. 1708890; Bio-Rad, Hercules, CA, USA) according to the manufacturer’s protocols. Real-time qPCR was performed in triplicate using the CFX96 Real-Time System (Bio‐Rad). Each 10-µl reaction consisted of 2 µl cDNA, 5 µl qPCR GoTaq^®^ Master Mix (cat. A6002; Promega, Madison, WI, USA), 1 µl of 3 µM mix of forward and reverse murine primers (**Supplementary Table S4B**), and 2 µl RNase-free water. The target gene expression levels were normalized to two housekeeping genes (*Hprt1, Sdha*, and *Tbp*).

### Histology and immunofluorescence staining

Standard hematoxylin (cat. X903; Roth) and eosin staining (cat. HT110232; Sigma, St. Louis, MO, USA) was performed for histomorphological analysis of the colon and the ileum (24). For immunofluorescence (IF) staining, paraffin sections were rehydrated (2 × 10 min Roti Histol, 2 × 5 min 100% EtOH, and 96%/90%/80%/70%/50% EtOH each 5 min) and washed for 5 min with distilled water and 0.1% Tween-20 (cat. 9127; Roth) in Tris-buffered saline (TBS-T). Antigen retrieval with citrate buffer for 20 min in a 98°C water bath was followed by blocking in 10% donkey serum (cat. ab7475; Abcam, Cambridge, UK) for 30 min. Primary antibodies were incubated at 4°C overnight, the slides washed with TBS-T, and then incubated with secondary antibodies and DAPI (cat. 567 D9542; Sigma) for 1 h (**Supplementary Table S4C**). After washing, the slides were covered with Immu-Mount (cat. 10662815; Epredia, Portsmouth, NH, USA). Images were acquired using Zeiss Axio Observer 7 and ZEN 3.5 software and were analyzed using ImageJ 2.1.

### Mouse grimace scale

A representative video was captured from mice treated for 5 days with 3% DSS or 3% DSS combined with 2 mg/kg amantadine. Control mice were either untreated or received an i.p. injection of 0.5 ml/kg carbon tetrachloride (CCl_4_) (cat. 289116; Merck, Darmstadt, Germany). The use of CCl_4_ in C57BL/6J wild-type mice was part of a separate project aimed at inducing liver fibrosis, and no additional animals were harmed for pain measurements in this study (LaGeSo ethics approval no. G0310/19). The videos and representative images were captured immediately after DSS treatment ended or directly following the i.p. injection, as CCl_4_ is known to induce moderate pain (36). Mice were filmed in their own cage and were observed for at least 10 min. Pain behavior was scored based on five categories of the MGS (i.e., orbital tightening, nose and cheek bulging, ear position, and whisker change) on a three-point scale from 0 to 2 (total score of 10) (27).

### Gene set enrichment analysis

To identify the pathways affected by amantadine, known chemical–target interactions for amantadine (PubChem CID 2130) were retrieved from PubChem (18). Of the 33 items listed, 30 are human genes (**Supplementary Table S5**). After removal of duplicates (*n* = 4) and the non-human (rat; *n* = 1) and virus-related genes (*n* = 2), 26 unique genes remained. These were subjected to gene set enrichment analysis using Enrichr (37). Significantly enriched terms (adjusted *p*-value <0.05) from the Molecular Signatures Database Hallmark 2020 were selected for further validation by qPCR in untreated and amantadine-treated colon samples.

### Quantitation and statistical analyses

Sample size was determined prior to the start of the experiments using G*Power 3.1 in consultation with the Institute of Biometry and Clinical Epidemiology at Charité and was approved by LaGeSo. The data distribution and variance assumptions were evaluated prior to statistical testing. For comparisons between two groups, an unpaired *t*-test was used. One-way analysis of variance (ANOVA) was performed for comparisons involving three or more groups, followed by Tukey’s multiple comparisons test. Statistical significance was defined as *p* < 0.05. Details on the sample size, replicates, and the statistical tests used can be found in the figure legends. The conditions of the experiments were known during data collection and analysis. GraphPad PRISM 9.5.1 was used for statistical analysis, and BioRender was used for the illustrations.

## Data Availability

The original contributions presented in the study are included in the article/**Supplementary Material**. Further inquiries can be directed to the corresponding author.
