# Supplementary material for: Deep learning, deeper relief: pipeline toward tailored analgesia for experimental animal models
Source: Front Immunol. 2025 Dec 12;16:1639881. doi: 10.3389/fimmu.2025.1639881 (PMC12741108; doi:10.3389/fimmu.2025.1639881)
Supplement: Supplementary file 1 [file DataSheet1.pdf]

Figure S2: PEDL⁺-selected analgesics show no interference with NF-κB signaling or immune cell composition in the small intestine

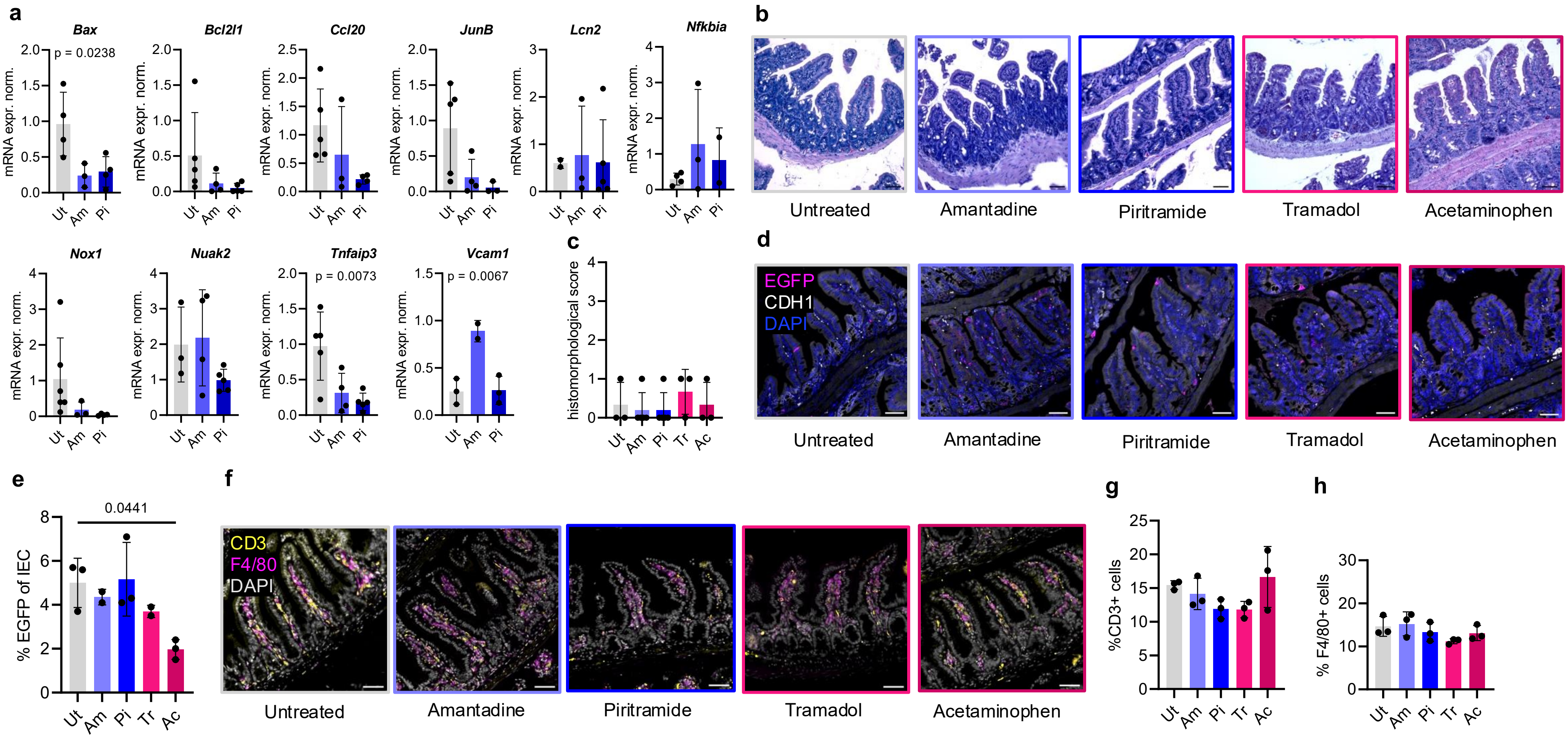

Figure S2: a. qPCR analysis of bulk colonic tissue of mice treated as in 2a. with targets of NF-κB. Normalized expression values (ΔΔCq) shown. Ut (Untreated), Am (Amantadine), Pi (Piritramide). Analysis: One-way ANOVA with Tukey's multiple comparison test, only significant p-values shown horizontally. **b**. H&E of ileum from mice treated with analgesics for 5 days or left untreated. Representative sections shown from at least n = 3 mice per group. Analysis as in a. Scale: 50µm. **c.** Histomorphological score from b. Analysis as in a. $F_{(4, 14)} = 0.4912$; $p = 0.7423$. Tr (Tramadol), Ac (Acetaminophen). **d.** Ileal immunofluorescence staining (IF) for EGFP and CDH1 from mice treated as in b. Representative sections shown from at least n = 2 mice per group. Scale: 50µm. **e.** Quantitation of c. shown as percentage of EGFP+ cells in ileal CDH1+ intestinal epithelial cells. Analysis as in a. $F_{(4, 8)} = 4.532$; $p = 0.0332$. **f.** Ileal IF for CD3 and F4/80 from mice treated as in a. n = 3. Scale: 50µm. **g.** Quantitation of f. shown as percentage of CD3+ cells in colonic lamina propria (LP). Analysis as in a. $F_{(4, 10)} = 2.288$; $p = 0.1315$. **h.** Quantitation of f. shown as percentage of F4/80+ cells in colonic LP. Analysis as in a. $F_{(4, 10)} = 1.707$; $p = 0.2243$.

Figure S3: Ileal differentiation of secretory cells is affected in piritramide and tramadol treated mice

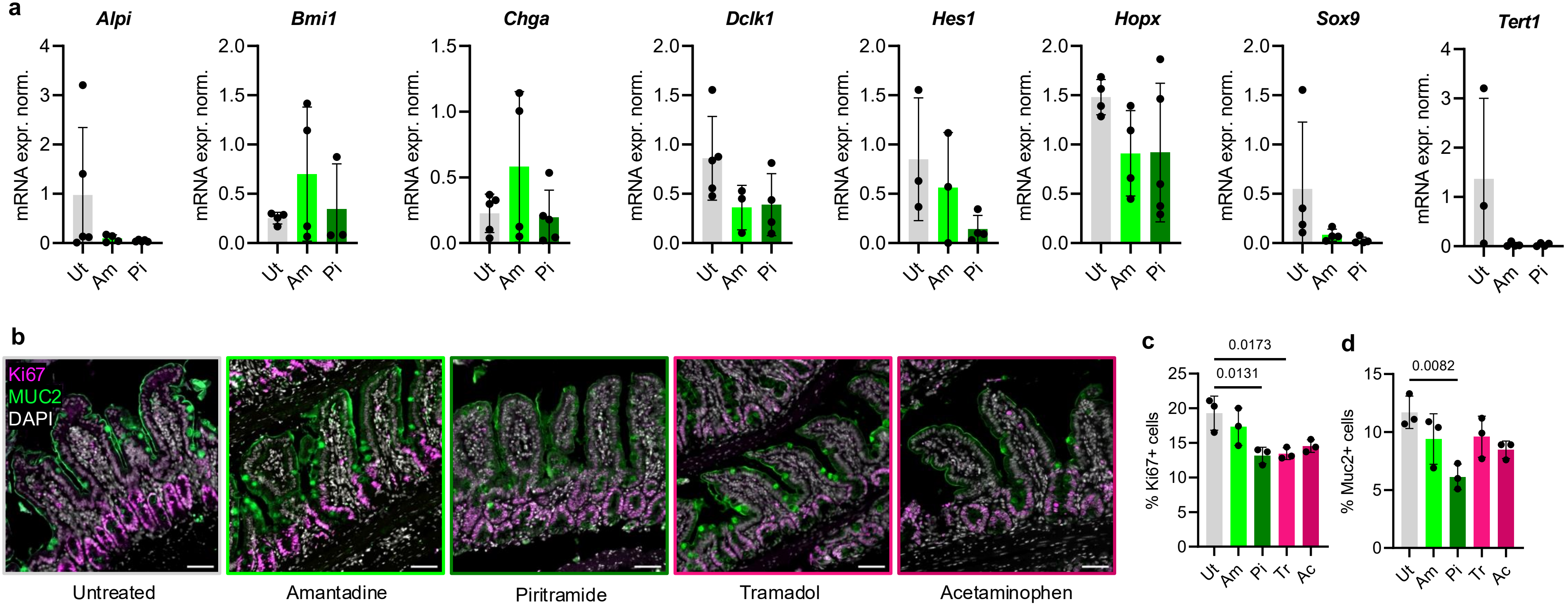

Figure S3: a. qPCR analysis of bulk colonic tissue of mice treated as in 3a. with markers of epithelial cell type and differentiation. Ut (Untreated), Am (Amantadine), Pi (Piritramide). Normalized expression values (ΔΔCq) shown. Analysis: One-way ANOVA with Tukey's multiple comparison test, only significant p-values shown horizontally. **b**. Ileal immunofluorescence staining (IF) for Ki67 and MUC2 from mice treated with analgesics for 5 days or left untreated. Representative sections shown from at least n = 3 mice per group. Scale: 50µm. **c.** Quantitation of b. shown as percentage of Ki67+ cells of intestinal epithelial cells (IECs). Tr (Tramadol), Ac (Acetaminophen). Analysis as in a. $F(4, 10) = 6.541$; $p = 0.0075$. **d.** Quantitation of b. shown as percentage of MUC2+ cells of IECs. Analysis as in a. $F(4, 10) = 5.255$; $p = 0.0153$.

Figure S4: Mouse grimace scale in validation of amantadine as analgesic

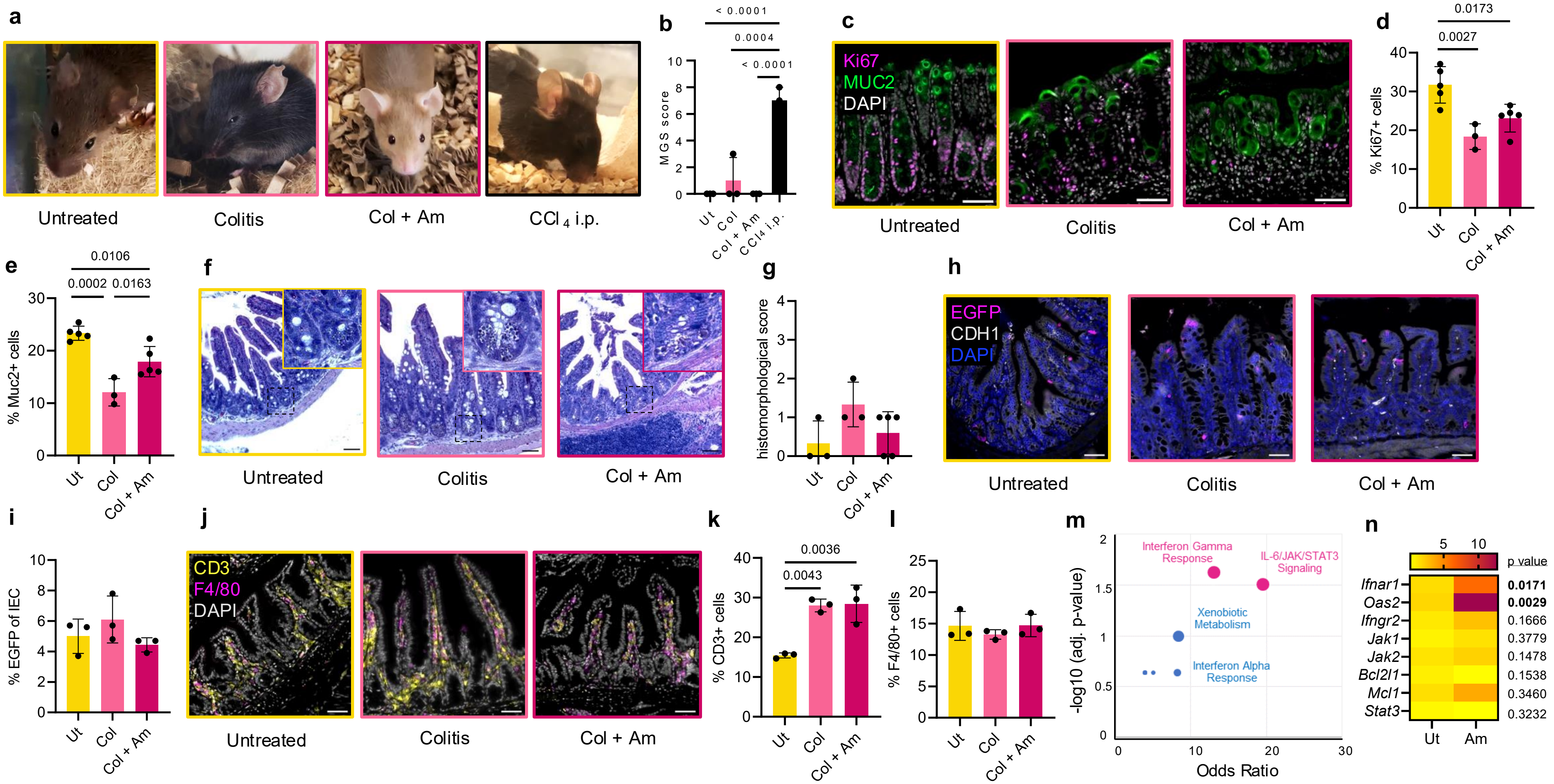

Figure S4: a. Images of mice treated as in Figure 4a. Control mice received an i.p. injection of carbon tetrachloride (CCl4). **b.** Quantitation of mouse grimace scale (MGS) score from a. Analysis: One-way ANOVA with Tukey's multiple comparison test, only significant p-values shown horizontally. $F (3, 8) = 34.00$; $p < 0.0001$. **c.** Colon immunofluorescence staining (IF) for Ki67 and MUC2 from mice treated as in a. Representative sections shown from at least n = 3 mice per group. Scale: 50μm. **d.** Quantitation of c. shown as percentage of Ki67+ cells of intestinal epithelial cells (IECs). Analysis as in b. $F (2, 10) = 11.61$; $p = 0.0025$. **e.** Quantitation of c. shown as percentage of MUC2+ cells of IECs. Analysis as in b. $F (2, 10) = 22.34$; $p = 0.0002$. **f.** H&E of ileum from mice treated as in a. Representative sections shown from at least n = 3 mice per group. Scale: 50μm. Insets show magnified views of dotted squares. **g.** Histomorphological score from f. Analysis as in b. $F (2, 8) = 2.603$; $p = 0.1347$. **h.** Ileal IF for EGFP and CDH1 from mice treated as in a. n = 3 mice per group. Scale: 50μm. **i.** Quantitation of h shown as percentage of EGFP+ cells from CDH1+ epithelial cells. Analysis as in b. $F (2, 6) = 1.677$; $p = 0.2639$. **j.** Ileal IF for CD3 and F4/80 from mice treated as in a. n = 3. Scale: 50μm. **k.** Quantitation of j. shown as percentage of CD3+ cells in colonic lamina propria (LP). Analysis as in b. $F (2, 6) = 19.61$; $p = 0.0023$. **l.** Quantitation of j. shown as percentage of F4/80+ cells in colonic LP. Analysis as in b. $F (2, 6) = 0.6489$; $p = 0.5557$. **m**. Scatter plot of terms from Molecular Signatures Database Hallmark 2020 using Enrichr, based on chemical-target interactions for amantadine (PubChem). Each point represents a single term, plotted by odds ratio and -log10(adjusted p-value). Significantly enriched terms with adjusted p-value < 0.05 in magenta. **n.** qPCR analysis of bulk colonic tissue of mice treated as in 2a. Normalized expression values (ΔΔCq) shown in heat map. Unpaired t-test. Significant p-values < 0.05 in bold.
